# Photobiomodulation Therapy and the Glymphatic System: Promising Applications for Augmenting the Brain Lymphatic Drainage System

**DOI:** 10.3390/ijms23062975

**Published:** 2022-03-10

**Authors:** Farzad Salehpour, Mahsa Khademi, Denis E. Bragin, Joseph O. DiDuro

**Affiliations:** 1 College for Light Medicine and Photobiomodulation, D-82319 Starnberg, Germany; farzadsalehpour1988@gmail.com; 2 ProNeuroLIGHT LLC, Phoenix, AZ 85041, USA; 3 Neurosciences Research Center (NSRC), Tabriz University of Medical Sciences, Tabriz 51666, Iran; drmahsakhademi@gmail.com; 4 Department of Neurology, University of New Mexico School of Medicine, Albuquerque, NM 87131, USA; dbragin@salud.unm.edu

**Keywords:** photobiomodulation, near-infrared light, glymphatic system, meningeal lymphatic vessels, amyloid-beta, neurodegenerative diseases, Alzheimer’s disease, Parkinson’s disease

## Abstract

The glymphatic system is a glial-dependent waste clearance pathway in the central nervous system, devoted to drain away waste metabolic products and soluble proteins such as amyloid-beta. An impaired brain glymphatic system can increase the incidence of neurovascular, neuroinflammatory, and neurodegenerative diseases. Photobiomodulation (PBM) therapy can serve as a non-invasive neuroprotective strategy for maintaining and optimizing effective brain waste clearance. In this review, we discuss the crucial role of the glymphatic drainage system in removing toxins and waste metabolites from the brain. We review recent animal research on the neurotherapeutic benefits of PBM therapy on glymphatic drainage and clearance. We also highlight cellular mechanisms of PBM on the cerebral glymphatic system. Animal research has shed light on the beneficial effects of PBM on the cerebral drainage system through the clearance of amyloid-beta via meningeal lymphatic vessels. Finally, PBM-mediated increase in the blood–brain barrier permeability with a subsequent rise in Aβ clearance from PBM-induced relaxation of lymphatic vessels via a vasodilation process will be discussed. We conclude that PBM promotion of cranial and extracranial lymphatic system function might be a promising strategy for the treatment of brain diseases associated with cerebrospinal fluid outflow abnormality.

## 1. Introduction

Photobiomodulation (PBM) therapy is the application of visible and near-infrared (NIR) light to stimulate cellular processes by changing biochemical activities of mitochondrial components at non-thermal and low-level doses [1]. Currently, PBM has obtained significant credibility and light medicine is fast becoming one of the most-accepted physical modalities. PBM has been demonstrated to be an effective approach for promoting cellular proliferation and microcirculation and for relieving pain and edema in various traumatic, acute, and chronic diseases [2]. Neuromodulation of the brain using transcranial and intranasal PBM has been shown to improve cerebral hemodynamics along with an increase in cerebral oxygenation and metabolic capacity [3,4]. Additionally, there is a rapidly increasing body of evidence to support that PBM therapy of the brain can ameliorate neuronal oxidative stress, neuroinflammation, and apoptosis, while promoting neurogenesis and synaptogenesis [5,6]. To date, no serious adverse effects have been reported in the literature for brain PBM therapy; however, caution must be considered with high-power laser sources (class 3B and 4) due to the hazard for macular lesions [7]. To deliver PBM to the brain, transcranial, intranasal, intraoral, intra-aural, and intravascular approaches have been proposed as non-invasive techniques to deliver photonic energy. Moreover, researchers have suggested that PBM therapy targeted at remote tissues (such as the abdomen or tibia) can produce indirect or abscopal effects providing neuroprotection to the brain via systemic mechanisms [8].

Recent evidence has suggested that the meningeal lymphatic vessels (MLVs) play a crucial role in maintaining brain homeostasis by draining macromolecules via both cerebral spinal fluid (CSF) and interstitial fluid (ISF) from the central nervous system (CNS) into the cervical lymph nodes (cLNs) [9,10,11]. An impaired cerebral lymphatic system is considered as a risk factor for neuroinflammatory diseases [12], neurovascular diseases [13], and impaired recovery from brain injuries [12]. In addition, preclinical studies have shown impaired meningeal lymphatic function in neurodegenerative diseases such as Alzheimer’s disease (AD) [9] and Parkinson’s disease (PD) [14]. In fact, impairment of MLVs function is a contributing factor in the development of AD and accelerates amyloid-beta (Aβ) aggregation [9]. Starting from 2018, a group of researchers from Russia has made efforts to test the modulatory actions of PBM therapy on the lymphatic drainage function of the brain. Their preliminary findings in a mouse AD model shed light on the potential effect of NIR PBM on the cerebral drainage system through the clearance of Aβ via MLVs [15]. Their follow-up studies also revealed that a PBM-mediated increase in the blood–brain barrier (BBB) permeability can result in further activation of the lymphatic clearance of Aβ from the brain. This action is most likely a result of PBM-induced relaxation of lymphatic vessels via a vasodilation process [16]. Further experiments outlined the beneficial effects of NIR PBM on the lymphatic clearance of blood products from the brain, an important strategy for the prevention of severe consequences after intracranial hemorrhage (ICH) [17].

In this review, we provide an overview of the brain lymphatic drainage system and its pathways. We also discuss the vital role of the lymphatic drainage system (cranial and extracranial lymphatics) in removing waste metabolites and toxins from the brain, maintaining CNS homeostasis and immune responses. We then review the recent animal research on the neurotherapeutic benefits of PBM on lymphatic drainage and clearance. Finally, we propose an underlying biological mechanism for the potential impacts of PBM on the cerebral lymphatic system and highlight this promising new therapeutic approach.

## 2. Brain Glymphatic Drainage System

### 2.1. The System, Its Components, and Pathways

Based on physiological findings of communication among different parts of the brain, the existence of a specific lymphatic drainage system in the brain of vertebrates has been suggested [18,19]. In 2012, Iliff et al., for the first time, identified a novel structure in the brain called the glymphatic system [20]. This system is considered as a crucial fluid-clearance system in the brain [21,22]. Studies on mouse models using different fluorescent tracers constructed this glymphatic drainage pathway in the brain [23,24].

**Figure 1:**
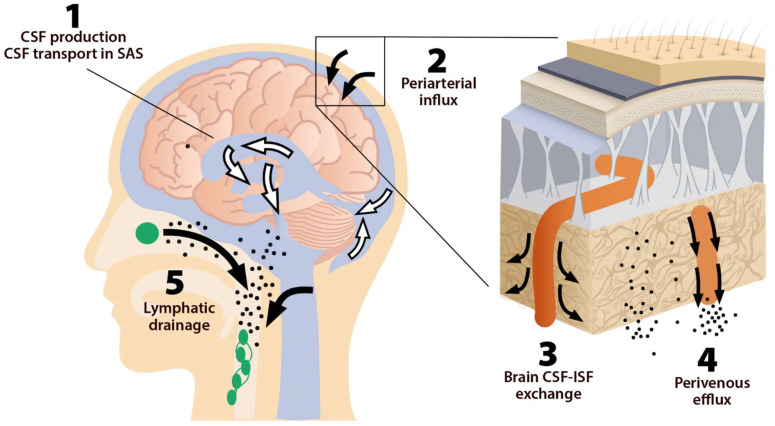
The five components of the glymphatic system. The fluid transport pathway is divided into five distinct segments: (1) cerebrospinal fluid (CSF) is produced by the choroid plexus and likely by extrachoroidal sources (capillary influx and metabolic water production); (2) arterial wall pulsatility drives CSF deep into brain along perivascular spaces; (3) CSF enters the brain parenchyma supported by aquaporin-4 (AQP4) water channels and disperses within the neuropil; interstitial fluid (ISF) mixes with CSF, (4) accumulates in the perivenous space, and drains out of the brain via (5) meningeal and cervical lymphatic vessels, as well as along cranial and spinal nerves Fluids from both the brain and the cribriform plate drain into the cervical lymphatic vessels, which then empty into the venous system at the level of the subclavian veins. The olfactory/cervical lymphatic drainage route is the primary bulk flow pathway.

**Figure 2:**
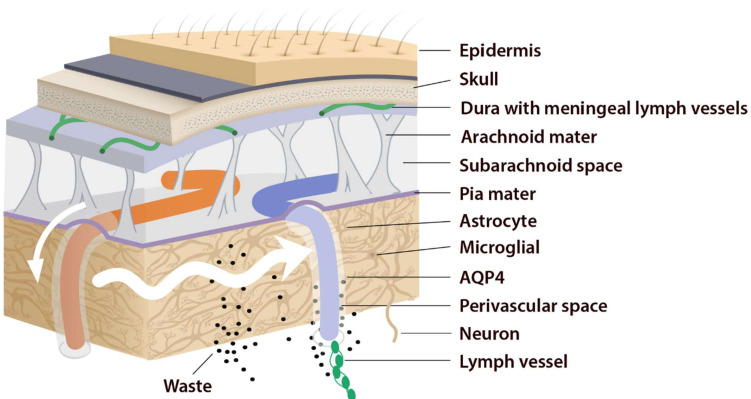
Periarterial influx of CSF into the brain tissue (small white arrow). CSF–ISF exchange supported by AQP4 channels in the vascular end feet plastered along the arterioles. From here, the fluid leaves the axons and moves towards the perivenous space in a path supported by astrocytes. Astrocytic AQP4 water channels facilitate this perivenous efflux of interstitial fluid, which drains to the dural lymphatic vessels.

This system consists of five main functional components, each facilitating the movement of CSF and ISF (Figure 1). The first compartment of the glymphatic system consists of the production of CSF by epithelial cells of the choroid plexus in the cerebral ventricles and circulation of CSF in the subarachnoid space, followed by the second, periarterial influx of CSF into the brain parenchyma. In fact, periarterial influx refers to the entrance of CSF into the periarterial spaces surrounding the arteries and its penetration deep into the brain tissue. Arterial pulsation caused by smooth muscle cells intensifies CSF movement inward along the periarterial space [25]. Exchange of CSF and ISF is the third component of this system, which occurs in the interstitial space of the brain parenchyma (Figure 2).

Astrocytes are believed to facilitate the fluid movement between periarterial spaces and the interstitium through water channels such as aquaporins-4 (AQP4) [20,26]. The fourth component is the glymphatic efflux, which consists of drainage of ISF into the perivenous spaces. The meningeal lymphatic system is the fifth component and final downstream clearance of the glymphatic system. MLVs drain waste products and other solutes from the CNS [9]. This ISF then flows towards the leptomeningeal arteries located at the cortical surface (sulci) and ultimately moves into the cervical lymphatics [20].

Indeed, this system was named “glymphatic” based on the involvement of glial cells “gl” and its similar function with the “lymphatic system” [27,28]. The brain glymphatic system has several essential physiological functions such as drainage of ISF from the parenchymal section of the brain to nearby lymph nodes. It is also involved in communication with the immune system, which regulates and monitors brain responses to neuroinflammation [29]. Moreover, the glymphatic system possesses numerous physiological functions in addition to solute clearance [30]. It is hypothesized that the glymphatic system has a role in rapid lipid transportation across the blood–brain barrier (BBB) and promote glial signaling [31]. Additionally, CSF is involved in the transportation of apolipoprotein E, essential for cholesterol transport, and most notably, synaptic plasticity [32]. CSF influx is also a vehicle for glucose and other vital nutrients that are necessary for the metabolism of astrocytes and neurons [30].

**Figure 3:**
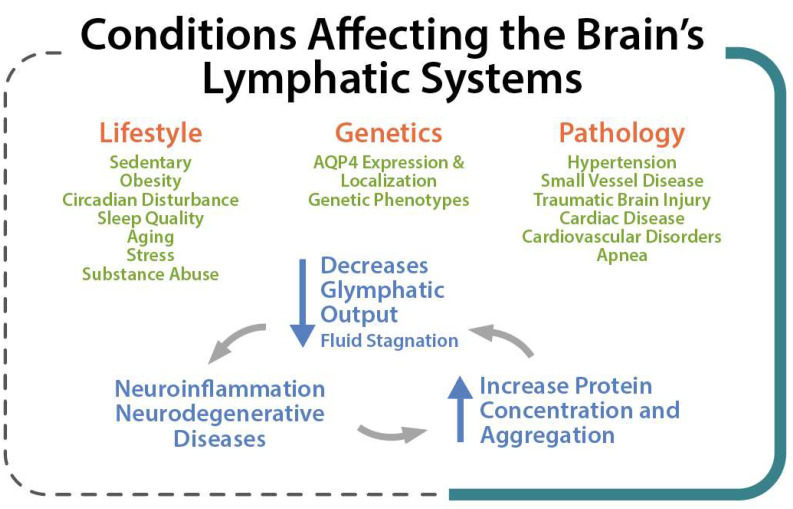
Lifestyle, Genetic and Pathological conditions that can strongly affect the brain lymphatic systems.

Lifestyle factors, genetics, and pathological conditions can modulate brain clearance and influence the risk of developing neurodegenerative diseases [33]. Several factors such as genetic phenotypes, body posture, aging, and the sleep–wake cycle could influence these physiological functions [23] so that an impaired cerebral lymphatic system is counted as a risk factor for neurodegenerative [34], neuroinflammatory [12], and neurovascular diseases [13] and tumors, as well as impaired recovery from brain injuries [12] (Figure 3). Pathological conditions can strongly affect the brain lymphatic systems. In various vascular disorders including hypertension, atherosclerosis, and small vessel diseases [35], any alteration in the composition of the constituent proteins can result in a significant decline in vascular plasticity and decrease cerebral blood flow (CBF) into the perivascular pathways. In arterial stenosis (either cervical or intracranial), blockage of CBF and obstruction of perivascular or paravascular channels are observed [13], leading to reduced ISF flow resulting in loss of CSF clearance from the brain. Glymphatic system dysfunction has been demonstrated to be associated with many neurological diseases such as AD and PD [14,28]. The glymphatic system has been described as the “final common pathway” for neurodegenerative diseases [36].

### 2.2. MLVs, Olfactory/Cervical Lymphatic Drainage Route, and Their Association with CSF Circulation

The absence of a conventional lymphatic vasculature in the CNS prompted a series of studies on rodents and human brains to identify MLVs as the lymphatic system of the CNS [37,38]. MLVs seem to provide a critical route for drainage of ISF and CSF. Various macromolecules and immune cells pass from CNS into the lymph nodes located in the deep cervical area [39,40,41]. More recently, strong evidence demonstrated that MLVs might be associated with the regulation of immune responses and also involved in the pathogenesis of neuroinflammatory diseases [42]. Animal studies have also shown impaired meningeal lymphatic function in AD [9] and PD [14]. In a neuroimaging study using a dynamic contrast-enhanced MRI, patients with idiopathic PD exhibited markedly decreased flow through the MLVs along the superior sagittal sinus and sigmoid sinus, as well as a significant delay in deep cLNs (dcLNs) perfusion [43].

Under normal physiological conditions, the olfactory/cervical lymphatic drainage route serves the primary bulk flow drainage pathway. The ethmoid bone and particularly the cribriform plate located at the anterior aspect of the brain (between the anterior cranial fossa and the nasal cavity) is considered a critical extracranial site of CSF outflow [44]. CSF in subarachnoid space passes through the cribriform plate along the olfactory nerves to the nasal lymphatics and cLNs. At the end of the route, CSF is deposited into the extracranial lymphatic system [13]. The continuous circulation and drainage of CSF are critical for removing CSF metabolic products and maintaining normal neural functions. The outflow routes of CSF are the arachnoid villi of the dural superior sagittal sinus [45], olfactory nerves, across the cribriform plate, and into the cervical lymphatic pathway [46].

**Figure 4:**
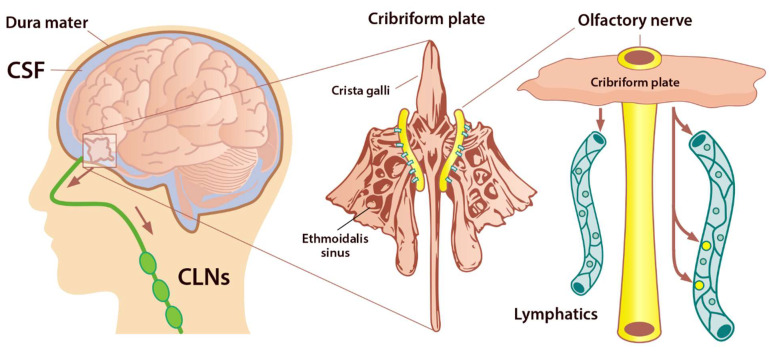
Perineural space surrounding olfactory nerve penetrates the nasal mucosa through the cribriform plate. The cribriform plate of the ethmoid bone is considered the key extracranial site of CSF outflow. CSF in SAS passes across the cribriform plate along olfactory nerves to nasal lymphatics and enters cervical lymph nodes (adapted from Semyachkina-Glushkovskaya et al. 2021).

The cribriform plate is a fenestrated bony plate of the ethmoid that separates the cranial and nasal cavities (Figure 4). Even though there are lymphatic vessels in the meninges [47], it has been demonstrated that CSF can drain through the cribriform plate in both humans and other mammals [48]. The main pathway by which CSF is removed from the skull is through the cribriform plate associated with the olfactory nerves [49]. The CSF is absorbed by lymphatic vessels located in the submucosa of the olfactory epithelium, in the nasal mucosa after passing the cribriform plate, and then drained into the cLNs. Any damage to the cribriform plate (by traumatic brain injuries or surgical methods) can lead to acute blockage of CSF outflow and, as a result, increase in resting intracranial pressure (ICP) and outflow resistance, emphasizing that the olfactory pathway represents the leading site for the CSF drainage [50]. There is a space between the olfactory sensory axons that provides a conduit for the outflow of CSF. Any damage to these nerves can also diminish the outflow of CSF through the cribriform plate [49] (Figure 4).

AQP are a family of small integral membrane proteins that significantly boost the permeability of cells to water and facilitate the movement of fluid down the pressure gradient in various tissues, including the brain [51]. So far, 13 AQPs have been found in mammals (AQP0–12). AQP1 maintains CSF production by the choroid plexus, and it is also expressed along the periphery of the olfactory bulb, nerve junction, and lining the foramina of the cribriform plate. Moreover, there are high levels of AQP1, 3, and 5 within the nasal cavity. These AQPs facilitate the flow of fluid out of the olfactory bulb and subarachnoid space into the nasal cavity via the extensive network of lymphatic vessels, which play an essential role in moving fluid throughout the body. AQPs are found in the meninges and at the cribriform plate and olfactory bulb junction [11]. These vessels crossing the cribriform plate play a key role in transporting fluid from the cranial cavity to the nasal cavity olfactory sensory nerves. Following CSF absorption by lymphatics, it is conveyed in larger ducts through numerous lymph nodes and eventually is deposited into the body’s lymphatic system. Evidence has also shown that aging decreases the elimination rate of CSF from the nasal/cribriform plate region [52,53].

### 2.3. Sleep and Clearance of the Brain

The glymphatic system uses convective flow between the CSF and ISF to remove toxic metabolites in/from the brain. CSF enters the brain parenchyma (functional parts) along a paraarterial route and exchanges with the ISF [54]. The ISF carries extracellular solutes from the interstitial (extracellular) space in the brain along paravenous drainage pathways (Figure 1). This activity is dramatically boosted during sleep and is related to increased interstitial volume, possibly by shrinkage of astroglial cells [55]. Emerging evidence shows that sleep is the primary driver of glymphatic clearance and is essential for the maintenance of brain function via the discharge of metabolites and neurotoxic wastes from the brain, which accumulates in the highly active brain during waking hours [36,56].

Comparing the brain ISF volume during deep sleep to wakefulness, the volume of the brain’s ISF increases by 40–60% [57]. Astrocytic AQP4 water channels that encircle the brain’s vasculature contribute to this increase in ISF. This increase in ISF is required for proper glymphatic function and facilitates the clearance of soluble proteins, waste products, and excess extracellular fluid. ISF increase leads to a 2-fold faster removal of neurotoxic waste products such as lactate and Aβ from the brain. This increase in the clearance of brain waste happens during non-rapid eye movement (NREM) sleep [58], and the majority of glymphatic activity occurs during deep, slow-wave sleep. Poor sleep quality and short sleep duration result in an increased amount of Aβ in the CSF as well as a risk of Aβ plaque formation [59]. In addition, tau levels have been shown to be increased in the ISF of the hippocampus following sleep deprivation [60]. Evidently, these neurobiological mechanisms can support the fact that neurodegenerative diseases such as AD, PD, Huntington disease, and frontotemporal dementias are strongly linked to sleep disturbances [61]. With the glymphatic system in mind, it is of interest to note that sleep quality decreases as a function of normal aging, and individuals over 60 years old rarely enter deep NREM (stages 3). The effectiveness of glymphatic fluid transport is directly linked to the prevalence of slow-wave activity. Therefore, the age-related impairment in sleep quality can cause a catastrophic drop in the clearance of brain waste and potentially increase the incidence risk of neurodegenerative diseases [36].

Recent evidence revealed that endocytosis occurs across the BBB during sleep, and inhibition of this process causes the need for more sleep [62]. In addition, several studies have reported that sleep deprivation can increase the activity of several pro-inflammatory mediators such as C-reactive protein, interleukin (IL)-1β, IL-6, IL-17, interferon-γ (IFN-γ), and tumor necrosis factor-alpha (TNF-α). These mediators suppress astrocytic maintenance of the BBB, causing an increase in its permeability [63,64]. Sleep deprivation has been shown to decrease influx efficiency along the perivascular space, thus impairing the function of the glymphatic system and disturbing AQP4 polarization in a mouse model [65].

## 3. PBM Therapy

PBM therapy or low-level light/laser therapy (LLLT) refers to the non-thermal application of visible and/or NIR light to stimulate biological processes [66]. Almost all PBM therapy procedures are applied in the wavelength range between 400 and 1300 nm from various light sources (e.g., lasers, light-emitting diodes (LEDs), or broadband light sources) [67]. One of the most recognized mechanisms for PBM has been suggested by a Russian photobiologist, Tiina Karu [68]. Her early work discovered that light-cell interaction probably should be considered as a light–mitochondria interaction [69]. Further studies with light at 600–850 nm wavelengths proved her hypothesis that the mitochondrial respiratory enzyme, cytochrome c oxidase (CCO), is the main photoacceptor responsible for the light absorption in the cells [70,71]. It is now believed that light energy is absorbed by metal centers of CCO, resulting in the excitation of electrons [72]. Along with this photoexcitation, nitric oxide (NO) is photodissociated from the CCO, leading to an increase in the mitochondrial membrane potential (MMP). This, in turn, promotes an increase in ATP production and modulates the levels of signaling molecules, including intracellular Ca^2+^ and reactive oxygen species (ROS), particularly the superoxide anion ($O_{2}^{-}$) and its stable product hydrogen peroxide (H_2_O_2_) [2]. As a secondary event, the above-mentioned primary responses change the intracellular redox potential, the intracellular pH, cyclic adenosine monophosphate (cAMP) levels, and expression of redox-sensitive factors such as nuclear factor kappa-B (NF-κB). Following this cascade of events, signal transduction processes induced by PBM will lead to activation of transcription factors and gene expression, which eventually will promote many biological functions such as cell metabolism, cell viability, proliferation, and differentiation [73,74,75].

Today, PBM is applied as a cutting-edge technology in numerous areas of medicine, such as wound healing, dentistry, muscle and tendon repair, dermatological conditions, and neurogenic pain [76]. In addition, recent research has focused on the application of PBM as a neuroprotective intervention for the treatment of CNS diseases [4,77], providing further breakthroughs in the field of neurorehabilitation. It has been shown that low levels of red/NIR light stimulate neuronal functions leading to neuroprotection and prevention of neuronal death, hypoxia, trauma, or neurotoxicity [4,6]. Transcranial PBM is the non-invasive delivery of light from external laser or LEDs sources (e.g., hand-held probes or wearable PBM helmets/headsets) to the head. This photonic energy is transferred onto subcranial tissues and to an extent, the cortical surface [4]. Intranasal PBM is another therapeutic approach delivering light energy through the nostrils, which has been shown to promote brain function in various CNS diseases such as depression, cerebral infarction, dementia, and Kleine–Levin syndrome [78,79,80,81,82,83].

### 3.1. Evidence on Potential Effects of PBM on the Brain Drainage System

Recently, a group of researchers from Russia have carried out a series of animal studies on the possible beneficial effects of PBM therapy on the lymphatic drainage function of the brain [15,16,59,84,85,86,87]. Their findings have opened up a new idea that PBM of the cranial and the extracranial lymphatics may be a promising approach for the treatment of brain disorders associated with CSF outflow abnormality [88]. Herein, we review their investigations and outline possible mechanisms of neurotherapeutic benefits of PBM on lymphatic drainage and clearance.

In 2019, they examined the idea that transcranial PBM might stimulate lymphatic drainage in an animal AD model by demonstrating improvement in the clearance of Aβ molecules from the brain following PBM therapy [15]. In the first step, they compared the effectiveness of four different skull fluencies (18, 25, 32, and 39 J/cm^2^) of 1268 nm laser on the reduction in Aβ accumulation in the brain. The skull fluence of 32 J/cm^2^ (cortical fluence of 4 J/cm^2^) was selected as an optimal PBM fluence because it was not associated with an increase in a skull temperature or morphological alterations of the brain and was significantly effective for the reduction in Aβ depositions in the brain. Although 39 J/cm^2^ effectively decreased Aβ accumulation, it resulted in a dura mater and arachnoid membrane injuries as well as a scalp temperature rise of 2 °C. In the second step, they studied the development of AD following the injection of Aβ in the hippocampus of mice and evaluated the effects of PBM (32 J/cm^2^) on Aβ distribution in the brain. The confocal microscopic analysis showed that PBM actively decreased the density of small Aβ plaques, whereas the density of large Aβ plaques did not differ between the PBM and the untreated group. The accumulation of Aβ in the brain of PBM-treated mice was also accompanied by the appearance of Aβ plaques in the dcLNs, compared with the untreated group. In the third step, they explored the PBM effects on clearance of gold nanorods (GNRs) from the brain into cervical lymphatics using optical coherence tomography (OCT) in vivo to monitor the rate of GNRs accumulation into the right dcLN. OCT data showed that PBM-activated clearance of GNRs was higher for treated mice. Clearance from the cortex, lateral ventricle, cisterna magna, and hippocampus was higher in the treated mice by 3.7-, 3.9-, 6.7-, and 9.3-fold, respectively. The results of atomic absorption spectroscopy (AAS), which exhibit the level of GNRs in dcLN, were also correlated with OCT data suggesting that PBM significantly increased the clearance of GNRs from both deep (hippocampus and ventricles) and superficial (cisterna magna and cortex) regions of the brain. Results from the neurological status (tested by startle reflex, round stick balancing, and beam walk tasks) and the neurobehavioral outcomes (tested by novel object recognition task) also showed an improvement in the PBM-treated mice compared with AD mice [15].

Given the fact that disruption of MLVs is an aggravating factor in the development of AD and promotes Aβ deposition in the meninges, in 2020, they investigated the potential benefits of PBM on lymphatic pumping and contractility, which are considered the main physiological mechanisms underlying fluid transport and waste clearance from tissues [59]. They started to test the hypothesis that PBM-promoted relaxation of lymphatic vessels via vasodilation might be one of the underlying mechanisms for increasing the permeability of lymphatic endothelium, thereby allowing larger molecules to be transported through to the lymphatic vessels. Their preliminary data revealed that low PBM fluencies (5 and 10 J/cm^2^) induced relaxation of the mesenteric lymphatics (extracranial and/or abdominal lymph vessels) in both systole and diastole with a decrease in contraction amplitude (with maximum response to 10 J/cm^2^). These low fluencies relaxed the lymphatic vessels, while higher fluencies of 30 and 70 J/cm^2^ completely blocked the contractility of the vessels. Their OCT imaging results also showed an increase in the diameter of the MLVs in systole and diastole following transcranial PBM. Data also demonstrated dilation of the MLVs and an increase in the number of macrophages inside the cavity of the vessel after transcranial PBM, most likely due to an increase in uptake of ISF (lymph). In the next phase, they studied the effects of the transcranial PBM (skull fluence of 64 J/cm^2^) on the drainage function of MLVs by analyzing the clearance of GNRs from the mouse brain. They injected GNRs in different brain regions as in their previous pilot study [15] (cortex, cisterna magna, right lateral ventricle, and hippocampus) and monitored the accumulation of GNRs in dcLN before and after transcranial PBM using OCT in vivo. PBM increased clearance of GNRs from the cortex to the dcLN by 55.7-fold. From the hippocampus, cisterna magna, and left ventricle, the clearance of the GNRs to the dcLN was also higher: 14.78-fold, 4.8-fold, and 2.3-fold, respectively. Their findings provided promising evidence that transcranial and remote PBM can augment the drainage and clearance function of MLVs, providing a therapeutic target for neurological disorders such as stroke and brain trauma, as well as preventing or delaying neurodegenerative diseases [59].

In their third study [87], they explored the effects of transcranial 1268 nm laser PBM on (1) clearance of two different tracers (GNRs and Evans blue dye (EBD)) from the brain via meningeal lymphatic system into the peripheral lymphatic system and (2) on the mesenteric lymphatics permeability of the mice. Their preliminary data showed that a cortical fluence of 9 J/cm^2^ (skull fluence of 32 J/cm^2^) exhibited a better stimulation of clearing function of MLVs than 2 and 5 J/cm^2^. Similarly, OCT data from the 9 J/cm^2^ group showed a gradual increase in the speed of GNRs accumulation in dcLN after its injection into the cisterna magna. PBM-mediated dilation of mesenteric lymphatics vessels was also associated with the decrease in resistance to the lymph flow. In the next phase, to better understand the mechanisms underlying the impacts of PBM on the lymphatic vessels, they investigated the effects of 9 J/cm^2^ light on the lymphatic permeability to immune cells such as macrophages. The results revealed that PBM can promote migration of macrophages from the lymphatic vessels into surrounding tissues, most likely through the decrease in transendothelial electrical resistance (TEER) integrity and an expression of tight junction (TJ) proteins (e.g., CLND, VE-cadherin, and ZO-1) [87].

In the fourth study, they aimed to test the hypothesis that the PBM-mediated opening of BBB might be one possible mechanism for the activation of Aβ clearance from the brain in AD mice [16]. First, they studied the effects of 1267 nm PBM with a cortical fluence of 9 J/cm^2^ (skull fluence of 32 J/cm^2^) on Aβ clearance from the mice brain using the immunohistochemical analysis of Aβ in the dcLNs. PBM-treated mice showed a pronounced Aβ level in the dcLNs, indicating the efficiency of the PBM for stimulation of Aβ clearance from the brain. The quantitative analysis also confirmed these results by representing a higher signal intensity from immunopositive Aβ plaques in the dcLNs in the PBM-treated mice. Their findings uncovered the lymphatic pathway of Aβ clearance from the brain which was also associated with the enhancement of the neurobehavioral status in AD mice. Their follow-up experiments showed that a PBM-mediated increase in the BBB permeability and subsequent increase in Aβ leakage occurs most likely as a result of PBM-induced decrease in transendothelial integrity and decrease in the expression of TJ proteins (e.g., CLND, VE-cadherin, and ZO-1) [16].

Their fifth study examined the lymphatic pathway of red blood cells (RBCs) clearance from the brain after intraventricular hemorrhage (IVH). They investigated whether transcranial PBM can improve RBCs evacuation from the ventricles to enhance the outcome after IVH [17]. First, using immunohistochemical and confocal colocalization analysis of the mouse and human brain samples (the next day after death due to IVH), they showed that RBCs moved from the ventricles into dcLNs via MLVs, confirming the lymphatic clearance of RBCs from the brain in the post-hemorrhagic period. They then studied the efficacy of transcranial 1267 nm PBM with a cortical fluence of 9 J/cm^2^ (skull fluence of 60 J/cm^2^) for stimulation of lymphatic clearance of tracers (GNRs and EBD) from the right lateral ventricle, mimicking the pathway of RBCs elimination from the brain of naïve mice. The transport of GNRs and EBD into dcLNs after its intraventricular injection was higher in the PBM-treated mice by 1.4- and 2.6-folds, respectively. In their follow-up experiment, they directly evaluated the effects of PBM on RBCs clearance from the mice brain after IVH and found that the number of RBCs transported into the dcLNs was significantly greater in the PBM group. They postulated that PBM facilitated RBCs drainage from the fluid-filled ventricles into the subarachnoid space where the RBCs are transported into the MLVs. This was accomplished by the PBM-mediated change in tone of the MLVs. In the final step, 3 days after surgical injection of blood into the right lateral ventricle, they treated mice with PBM 3 times in 7 days with a cortical fluence of 9 J/cm^2^. They found that PBM contributed to a faster recovery of ICP after IVH, along with a 1.57-fold decrease in mortality and a significant reduction in the level of stress [17].

In the last and most recent study on AD mice [84], they tested the hypothesis that transcranial PBM stimulates Aβ clearance from the brain through the activations of cerebral lymphatic drainage and probably via an increase in cerebral energy metabolism. Application of transcranial 1268 nm laser PBM with a cortical fluence of 4 J/cm^2^ (skull fluence of 32 J/cm^2^) for 9 days significantly reduced Aβ plaques in the brain of AD mice along with a significant increase in clearance of Aβ via MLVs. Their further investigation shed light upon the possible involvement of PBM-induced improvement in blood oxygen saturation (SpO_2_) of the brain on the stimulation of lymphatic Aβ clearance. They suggested that an increase in oxygen saturation leads to improved mitochondrial ATP production that can stimulate lymphatic contractility leading to increased drainage and clearing functions of the meningeal lymphatic system [84].

### 3.2. PBM and Nitric Oxide

Studies have shown increased blood flow during and after PBM both in animal models and in humans [89,90]. However, a critical question regarding this escalation of blood flow has remained open. Does this increased blood flow arise from the PBM-mediated production of NO? If so, what is the actual source? Is it NO that is photodissociated from hemoglobin in circulating RBCs, or NO that is photodissociated from other labile NO stores in the blood vessel wall, or is it derived from the dissociation of NO that has bound to CCO in the mitochondria? Nevertheless, it has been proposed that red/NIR light appears to be best for dissociating NO from CCO, thereby reversing the signaling consequences of excessive NO binding [2].

Impaired cerebral vascular perfusion has been widely known as one of the early manifestations in most of the CNS diseases [91]. Animal research has shown that PBM can improve neuronal NO levels and CBF, resulting from activation of endothelial NO synthase (eNOS) protein [90], and also can increase the blood vessel diameter [92]. It is also suggested that PBM can affect the regional CBF, most likely mediated by NO and glutamate [90]. Uozumi et al. found that the transient CBF improvement by PBM was dependent on the NOS activity and NO levels as well. They showed that transcranial 808 nm laser PBM of the naïve mice increased cortical NO levels (by 50%) immediately after starting the PBM, and gradually improved CBF in the PBM-exposed (by 30%) and the opposite hemisphere (by 19%) at 45 min after starting the irradiation [90].

PBM has also been shown to improve endothelial function through the activation of cellular pathways responsible for the modulation of inflammation and angiogenesis, as well as vasodilatation [93]. NIR light at 890 nm could significantly increase NO levels (with a peak at 5 min post-irradiation) in venous blood in healthy individuals [94]. Endothelial NOS (eNOS) is found in endothelial cells, which are the cells that line the inner surface of blood vessels as well as lymph ducts. eNOS can be activated by the pulsatile flow of blood through vessels, leading to a “shear stress” on the membrane of the endothelial cells as the column of blood in the vessel moves forward and then stops. Indeed, NO produced by eNOS maintains the diameter of the blood vessel (vasodilation) so that perfusion of various tissues (skin, bone, muscle, and nerves) is maintained at optimal levels. This eNOS-mediated NO can also activate the growth of new blood vessels (angiogenesis). NO relaxes smooth muscle cells and therefore dilates resistance vessels and lymphatics, leading to an increase in blood supply for repairing tissues and removal of the damaged cells [95]. In fact, increased lymphatic flow removes metabolic waste products and reduces edema. It has been shown that NO can be produced enzymatically following an increase in NOS activity after PBM, possibly by elevating intracellular Ca^2+^ levels [2,96].

As discussed before, it has been reported that PBM-induced relaxation of the mesenteric lymphatics endothelium is accompanied by increased permeability of lymphatic walls as well as a decreased expression of TJ proteins [87]. The TJ proteins are structural compounds of mature lymphatic vessels and play an essential role in moving ISF and immune cells through the lymphatic endothelium [88]. In fact, increased permeability of lymphatic endothelium is the main mechanism allowing for toxins to be transported by the collecting lymphatics, contacting local immune cells to activate immune responses. These effects might be related to a PBM-mediated increase in the eNOS activity. In other words, the PBM-mediated dilation of lymphatic vessels could be due to an increase in the eNOS activity [97]. Considering the fact that the improvement of endothelial NO production is a well-recognized mechanism of PBM [97] and that the lymphatic behavior is actively regulated by NO [98], it appears that PBM activates the NO synthesis in lymphatic endothelium isolated cells. Thus, the lymphatic vessel’s contractility may be the possible underlying mechanisms for PBM-induced lymphatic clearance of macromolecules from the brain [88].

### 3.3. PBM and Neuroprotection

Researchers have explored the neuroprotective effects of PBM against toxicity induced by Aβ [99,100,101,102]. A series of studies conducted by Da Xing’s research team has shown novel findings [99,100,102]. First, they proposed that activated Akt induced by laser PBM (632.8 nm, 2 J/cm^2^ at culture surface) interacts with and then inactivates GSK3β upon Aβ_25-35_ treatment. Following this step, due to the inhibition of GSK3β, β-catenin accumulates in the cytoplasm and then translocates into the nucleus. Subsequently, it acts as a transcriptional cofactor to improve neuronal survival [99]. Then in the follow-up research using the same laser source [100], they showed that 2 J/cm^2^ protects SH-SY5Y cells against Aβ_25-35_-induced toxicity only at 24 and 48 h post-PBM. After exposure to Aβ_25-35_, the cell viability of both SH-SY5Y cells and hippocampal neurons was increased by PBM in a dose-dependent fashion, representing a significant increase only at the 2 and 4 J/cm^2^. Their results also revealed that 2 J/cm^2^ was enough to protect hippocampal neurons against Aβ_1-42_ toxicity [100]. Recently, Da Xing and colleagues found that by increasing the mitochondrial CCO activity and thereby increasing the levels of cAMP and ATP, laser PBM (632.8 nm, 2 J/cm^2^ at culture surface) can activate the PKA/SIRT1 signaling pathway in SH-SY5Y-APPswe cells, leading to decreased Aβ levels [102]. Duggett and Chazot have also proved the neuroprotection effects of 1068 nm wavelength, proposing that a 4.5 J/cm^2^ can protect CAD neuroblastoma cells from Aβ_1-42_-induced cell death [101]. A 1070 nm PBM (4.5 J/cm^2^ at scalp surface with 10 Hz pulsed mode) has also been reported to decrease cerebral Aβ levels and therefore enhance cognitive performance in AD mice through microglia activation and promotion of angiogenesis. NIR PBM could trigger microglia rather than astrocyte responses with a change in morphology and increased colocalization with Aβ. The response of microglia to PBM was negatively correlated with the Aβ level, proposing that PBM decreases the Aβ deposition, probably via eliciting microglia activation and recruiting microglia to Aβ burden. The perivascular microglia were also decreased after PBM therapy, whereas an increase in cerebral vessel density was observed in PBM-treated AD mice. This increase in vessel density was positively correlated with clearance of Aβ burden, indicating that 1070 nm PBM can diminish Aβ deposition most likely through increasing cerebral vessel density [103]. In addition to these, 630 nm laser PBM therapy has been shown to decrease Aβ-disrupted flow of ISF by smashing Aβ deposition in the extracellular space and thereby reversing cognitive impairments in an APP/PS1 mouse model of AD [104].

### 3.4. Intranasal and Systemic PBM Therapies and Their Effects on the Brain Drainage System

Considering the fact that the blood capillaries are abundant in the nasal cavity and the blood flow is relatively slow, it is postulated that systemic effects on hematologic cells in the blood would contribute to the neuro-therapeutic benefits reported by the intranasal PBM technique [78,81]. Intranasal PBM has been shown to increase CBF [80], decrease blood viscosity [105], increase hemorheology [105], and increase blood coagulability status [106]—enhancements in blood rheology that are linked to improved cognitive functioning [107] and mood [108]. With respect to the brain drainage system, intranasal PBM might also serve as an effective treatment for obstruction of the cribriform–lymphatic route, thereby improving the CSF outflow. This is thought to occur because PBM-induced NO can modulate the lymphatic vessels contraction and subsequent increase in lymph flow [88,109]. Given this, it is speculated that the modulation of blood circulation and drainage function of the lymphatic system might be the underlying action mechanisms of intranasal PBM in the previously shown studies on dementia and other neurodegenerative diseases [78].

Recently, the possibility that PBM therapy targeted at a remote tissue (e.g., abdominal tissue) might elicit systemic mechanisms that provide neuroprotection of the brain is of great interest to researchers [83,110,111]. Today, there are a number of convincing examples of the possible systemic effects of PBM in animal models of AD [112,113] and PD [74,114,115]. Although the mechanisms underlying the phenomenon of systemic or indirect effects of PBM are not clear, we propose that the effect of PBM on the lymphatic system could be one of them. As discussed above, 1267 nm laser PBM of the mice’s abdominal region results in a relaxation of the mesenteric lymphatics with a decrease in contraction amplitude leading to subsequent increases in the clearance of GNRs from the brain, partly via MLVs [59]. PBM therapy targeted to the abdominal region has also been suggested to change the gut microbiota—with the release of yet unidentified circulating mediators—promoting a neuroprotective action on the brain [113]. It is also thought that bone marrow-derived stem cells—in particular, mesenchymal stem cells—can drive remote PBM-induced neuroprotection on the brain tissue [116,117], as they can easily transmigrate across the BBB [115,118].

## 4. Conclusions

Emerging evidence suggests that the MLVs play a key role in maintaining brain homeostasis by draining macromolecules via both CSF and ISF from the CNS into the cLNs. An impaired cerebral lymphatic system is considered a risk factor for neuroinflammatory diseases, neurovascular diseases, and impaired recovery from brain injuries. Animal research also demonstrates impaired meningeal lymphatic function in AD and PD. In particular, MLVs dysfunction can accelerate the development of AD through Aβ aggregation. Together, PBM-mediated promotion of glymphatic and extracranial lymphatic system function might be a promising candidate for the treatment of various brain diseases associated with CSF outflow abnormality. Because of good penetration onto the subcranial, brain cortex, and even into subcortical structures, transcranial PBM can stimulate MLVs, which might be one of the mechanisms underlying the positive clinical outcomes of PBM with neurodegenerative conditions [119,120]. Transcranial PBM can affect the aqueous component of the CSF/ISF structure because of light’s effect on the structure of the water molecules, creating a freer-flowing, slippery effect. Another factor is PBM-induced production of NO that can increase overall blood perfusion, increasing ISF/CSF diffusion components.

It should be noted that the therapeutic effects of transcranial PBM on subcortical regions of the human brain using biomodulatory wavelengths (e.g., 600–1300 nm) are not well understood. This is due, in part, to the poor penetration of red and NIR light through the skull/scalp into the deep brain areas. Given this, delivery of sufficient light dose to deeper structures in the human brain is still a challenge in the transcranial PBM field. Recently, nanoparticle engineering, in combination with biophotonic techniques, has been suggested as a way to overcome this problem. Considering the fact that photons in the third optical window (1550–1870 nm) have the highest penetration rate into brain tissue, it is speculated that upconverting nanoparticles (UCNPs) could help in delivering light to the deep brain by converting these photons to visible and NIR spectrum with higher energies and a greater biological effect. Of note, UCNPs exhibit good ability to cross the BBB, and also their low toxicity makes them a promising candidate for application in brain disorders.

Considering potential applications in human subjects, it is speculated that transcranial PBM can create an increase in surface temperature on the skull, thereby increasing the temperature gradient, creating a heat sink of blood to cool the sleeping brain, if and when the transcranial irradiation of the light is conducted immediately in bed before sleep. Intranasal PBM can also be an effective non-invasive approach for the treatment of cribriform plate obstruction, which is the main reason for the development of various brain pathologies due to the blocking of CSF drainage. Intranasal PBM reduces the viscosity of the blood. In turn, this would create less stacking of RBCs (rouleaux formation), which would allow a better flow of microcirculation through the areas that are smaller than capillaries. In addition, it has been suggested that brain cells might also benefit indirectly/systemically from the PBM of circulating blood or different underlying organs. A systemic PBM application using body pad LED devices across the carotid and or vertebral arteries can facilitate vasodilation and increase blood flow to the brain parenchyma in humans. Body pad-style applications of LEDs over the corpus and or cervical lymph tissue can presumably also increase the activity of the dcLNs and act as a pulling aspect of glymphatic flow. Applying PBM on the chest or abdomen area may prove effective in stimulating the mesenteric lymph vessels to augment lymphatic pull. Finally, PBM being a light energy-based therapy, has a circadian influence, so the timing of when to apply the red/NIR light is even more critical with a PBM intervention of the glymphatic and meningeal lymphatic systems. We can only speculate that future research will clarify the photonic impact of PBM on each of the five components of the glymphatic system.

## Data Availability

Not applicable.
